# Resting‐State Coactivation Patterns of Language Reorganization in Brain Tumors

**DOI:** 10.1155/np/1421115

**Published:** 2026-05-21

**Authors:** Antonio Napolitano, Leonardo Spitoni, Mehrnaz Jenabi, Kyung K. Peck, Andrei I. Holodny, Jennifer Moliterno, Todd Constable, Luca Pasquini

**Affiliations:** ^1^ Department of Radiology and Biomedical Imaging, Yale School of Medicine, New Haven, Connecticut, USA, yale.edu; ^2^ Medical Physics Department, Bambino Gesù Pediatric Hospital, Rome, Italy; ^3^ Radiology Department, Memorial Sloan Kettering Cancer Center, New York City, New York, USA, mskcc.org; ^4^ Department of Neurosurgery, Yale School of Medicine, New Haven, Connecticut, USA, yale.edu

**Keywords:** brain tumors, coactivation pattern analysis, functional MRI, language reorganization, resting-state fMRI

## Abstract

We investigated the temporal dynamics of resting‐state brain activity associated with language reorganization in patients with left‐hemisphere brain tumors using coactivation pattern (CAP) analysis of functional MRI. This retrospective study included 106 right‐handed patients who underwent both resting‐state and task‐based fMRI before surgery. Language dominance was determined using a phonemic fluency task, and CAP analysis identified recurring whole‐brain activation patterns. Dynamic properties of CAPs, including persistence, transitions, in‐degree, and out‐degree, were compared between patients with typical and atypical language dominance and between those with and without postoperative aphasia. Six distinct CAPs corresponding to known functional networks were identified. Patients with atypical language dominance showed higher out‐degree (*p* = 0.004) and transitions (*p* = 0.006) in the dorsal default mode network (DMN; CAP2) and lower persistence in the visuospatial network (VSN; CAP3; *p* = 0.002) compared with patients with typical dominance. Patients without postoperative aphasia demonstrated higher transitions in VSN CAP6 (*p* = 0.024). Logistic regression analyses were performed to evaluate the prediction of postoperative aphasia. A model including CAP metrics and the language laterality index (LI) demonstrated good predictive performance (area under the curve [AUC] = 0.851, accuracy = 82.4%, sensitivity = 71.9%, and specificity = 88.1%), outperforming a model based on LI alone (AUC = 0.640 and accuracy = 67.0%). No significant differences were observed by tumor type or volume. These findings indicate that CAP dynamics capture distinct neural states associated with language dominance and postoperative outcomes. Specific CAP metrics may serve as potential imaging biomarkers of functional reorganization, supporting prognostic evaluation and surgical planning in patients with brain tumors.

## 1. Introduction

Brain tumors can alter brain function both locally and at a distance, leading to widespread changes in functional networks [1–3]. These alterations can impair clinical performance [4], but also trigger compensatory mechanisms of neuroplasticity [5]. Prior work has shown that tumors can disrupt distant functional connections and degrade network architecture, including reduced segregation, clustering, and efficiency [6]. These effects likely impair information transfer across the brain, contributing to cognitive deficits. At the same time, evidence supports the brain’s ability to reorganize in response to tumor growth or resection, particularly in functions like language [7–10]. In patients with left‐hemisphere tumors, language processing often shifts toward peritumoral regions or the right hemisphere overtime [1, 2], suggesting dynamic reorganization. This phenomenon is clinically relevant, since it can affect the surgical approach and timing, the extent of resection, and the postsurgical prognosis [11]. When critical language areas shift away from the tumor, neurosurgeons may be able to perform a more extensive resection with reduced risk of impairing language function [12]. Conversely, if essential language regions remain close to or within the tumor margin, a more conservative approach may be warranted to preserve function. Currently, the indication of whether language function has successfully reorganized is often revealed postoperatively, through the presence or absence of new language deficits. Knowing the mechanisms of language reorganization is essential for tailoring surgical strategies and optimizing patient outcomes.

However, understanding this phenomenon remains a challenge. Currently, there are no reliable means to differentiate patients who undergo adaptive language reorganization, resulting in preserved or improved function, from those who develop maladaptive changes that may reflect inefficient or compensatory but ineffective activations. Prior studies highlighted than certain networks such as cognitive control network (CCN) or default mode network (DMN) may participate in language reorganization and facilitate preservation of speech in patients with brain tumors [13]. Despite these efforts, evidence remains limited. Resting‐state fMRI is a powerful tool to study brain connectivity [14], measuring spontaneous fluctuations in neural activity that follow reproducible patterns [15]. Conventional approaches average connectivity over several minutes, assuming stability overtime [15]. Yet cognition is inherently dynamic, and these methods may overlook transient, meaningful fluctuations that underlie different mental states [16]. Coactivation pattern (CAP) analysis addresses this limitation by identifying momentary patterns of whole‐brain activity and quantifying transitions between them [17]. By treating each fMRI frame as a unit of analysis, CAP provides a temporally resolved view of how the brain cycles through distinct functional states, such as those related to attention, motor, or language processing [16, 18]. Prior studies in healthy individuals have shown that CAPs reveal age‐related changes in flexibility across cognitive networks [19], but CAP has not yet been applied to investigate language network dynamics in patients with brain tumors.

In this study, we applied CAP analysis to resting‐state fMRI data from patients with left‐hemisphere tumors, aiming to compare the temporal dynamics of brain states between individuals with left language dominance vs. atypical dominance (right or codominant) as a surrogate of language reorganization. We also investigated whether specific CAP dynamics were associated with language performance, focusing on the presence or absence of aphasia after surgery. We hypothesized that patients with adaptive language plasticity would show greater engagement of high cognitive functions such as CCN and DMN.

## 2. Methods

### 2.1. Participants

This study received approval from the Institutional Review Board and was conducted using a retrospective cross‐sectional design. The requirement for informed consent was waived due to the nature of the study. All procedures adhered to the principles outlined in the Declaration of Helsinki. We retrospectively reviewed our institutional database to identify eligible patients between January 2016 and July 2025, applying the following inclusion criteria: newly diagnosed tumors located in the left hemisphere, presence of a single lesion (excluding cases with multiple lesions such as multifocal metastases), right‐handedness as determined by the Edinburgh handedness inventory, pathologically confirmed diagnosis, and availability of both task‐based and resting‐state fMRI acquired preoperatively in the same session. Cases were excluded if imaging data were compromised by artifacts related to tumor characteristics (e.g., large hemorrhagic components) or patient‐related factors such as motion. Both fMRI modalities were required for inclusion: task‐based fMRI was used to assess language laterality, which is considered more reliable with this approach, while resting‐state fMRI was necessary for subsequent network‐based analyses. Speech deficits were evaluated by an attending neurologist 1 week postoperatively and retrieved from patients’ clinical charts. The language assessment was designed to comprehensively evaluate key domains of expressive and receptive language. This included a structured examination of speech fluency, assessing spontaneous verbal output for ease, flow, and grammatical structure. Repetition tasks were administered to evaluate phonological processing, typically involving repetition of words, phrases, and complex sentences. Naming ability was assessed through confrontation naming tasks, where patients were asked to identify pictured objects or actions, probing lexical access and semantic retrieval. Comprehension was evaluated using both simple and complex verbal commands, as well as sentence‐ and paragraph‐level understanding, to examine receptive language function.

### 2.2. MRI Protocol

MRI was performed using a 3T scanner with 24‐channel head coils. fMRI was acquired with single‐shot gradient echo EPI (TR/TE = 2500/32 ms, section thickness = 4 mm, matrix = 64 × 64 mm, FOV = 240 mm, acquisition volume = 160, and scanning duration = 6 min, 55 s). fMRI coverage matching anatomical scans, including 2D FLAIR (TR/TE = 10,000/106ms, TI = 220 ms, matrix 256 × 256), 2D T1 postcontrast (TR/TE = 600/20 ms, matrix 256 × 256), and 3D T1‐weighted anatomic images using a spoiled gradient recalled‐echo sequence (TR/TE = 22/4 ms, matrix 256 × 256, section thickness = 1 mm) were acquired as routine clinical scans. During resting‐state fMRI acquisition, participants were asked to remain still, focus their gaze on a central fixation cross, and allow their minds to relax without engaging in structured thoughts. For the task‐based fMRI focused on language function, patients completed a phonemic fluency task delivered through a visual interface. In this task, they were instructed to silently think of as many words as possible starting with a given letter shown on the screen. Each block featured two different letters, each displayed for 10 s. The overall paradigm followed a block design, alternating between 20 s of active task performance and 40 s of rest.

Tumors were manually segmented by a fellowship‐trained neuroradiologist using the 3D Slicer segmentation tool27 (https://download.slicer.org/). Two ROI were labeled: (1) the area of contrast enhancement, segmented on postcontrast 3D T1‐weighted images, and (2) the area of edema/nonenhancing tumor, segmented on FLAIR images. Location labels were assigned automatically in our pipeline from the automated anatomical labeling (AAL) atlas28 (https://www.gin.cnrs.fr/en/tools/aal/) based on ROI 1 for HGG and metastases and ROI 2 for LGG. Seven main locations were studied, merging subareas from the AAL atlas: frontal lobe, parietal lobe, temporal lobe, occipital lobe, central area (basal nuclei), insula, and cerebellum.

### 2.3. Task‐Based fMRI Laterality Analysis

Task‐based fMRI data were analyzed using the Analysis of Functional NeuroImages (AFNI) software suite [20] (https://afni.nimh.nih.gov), which generated task‐related correlation maps. Preprocessing steps included correction for head motion via 3D rigid‐body alignment, spatial smoothing with a Gaussian kernel of 4 mm full width at half maximum (FWHM), and the removal of both linear trends and high‐frequency noise. To identify task‐related brain activation, statistical parametric maps were produced by cross‐correlating the observed signal time course with the modeled block design. Activation maps for the phonemic fluency task were thresholded at a correlation coefficient of *r* > 0.5 (uncorrected *p* = 2 × 10^−11^). To reduce the likelihood of false positives, voxels exhibiting a standard deviation greater than 8% of their mean signal intensity were excluded by setting them to zero. Language laterality was assessed using an hemispheric laterality index (LI), capturing activity within the whole hemisphere, as in prior work [21]. A threshold‐independent approach was employed to reduce interindividual variability, as described in earlier studies [3]. The LI computation followed a multistep procedure: (1) T1‐weighted anatomical and functional scans were coregistered to the MNI152 standard space (https://www.lead-dbs.org/about-the-mni-spaces/) using nonlinear registration via Advanced Normalization Tools (ANTs, https://stnava.github.io/ANTs/); (2) anatomical scans were parcellated using the AAL atlas; (3) the top 5% most active voxels on the correlation map were identified, and their mean value calculated; (4) voxels within each hemispheric ROI exceeding 80% of this mean were counted; (5) laterality indices were calculated using the standard formula: (*L* − *R*)/(*L* + *R*), where *L* and *R* represent the number of suprathreshold voxels in the left and right ROI, respectively. The hemispheric ROI excluded the cerebellum and primary visual regions (bilateral pericalcarine cortex, corresponding to areas V1 and V2). Patients were classified as atypical dominant (AD) if their LI was less than 0.2, and as left dominant (LD) if their LI was equal to or greater than 0.2 [22].

### 2.4. Resting fMRI Analysis

The resting state fMRI data was preprocessed using FMRIB Software Library for motion correction (MCFLIRT tool), spatial smoothing with 4 mm Gaussian kernel and skull‐stripping (BET tool). Using FSL and SPM12 (Statistical Parametric Mapping 12v. 7771), fMRI data of each subject was first spatially coregistered to T1 anatomical images (FSL Linear Registration) and then to the 152‐brain Montreal Neurological Institute (MNI) space (SPM12 normalization).

### 2.5. CAP

CAP analysis is a data‐driven method used to identify recurring brain states by examining the spatial distribution and magnitude of the blood oxygenation level‐dependent (BOLD) signal across fMRI volumes allowing to capture instantaneous brain configurations at single time points. The CAP analysis process typically involves three main steps. First, the signal intensity at each time point within each voxel, is transformed by subtracting the mean signal and then dividing by the standard deviation across all time points (i.e., mean equals to zero and standard deviation is equal to 1). Second, a clustering algorithm is applied to group the fMRI volumes, either all time points or a subset showing high activity in a predefined seed region, into clusters based on recurring CAPs observed across subjects and time. Clustering is a technique for categorizing objects into groups such that the similarity within each group is higher than between groups. CAP analysis usually applies the k‐means algorithm to classify fMRI volumes into *k* clusters based on their spatial similarity. Third, the fMRI volumes assigned to each cluster were averaged, producing *k* representative maps, which we refer to as CAPs [16, 23].

CAPs were extracted using the TbCAPs Toolbox [24] (https://doi.org/10.1016/j.neuroimage.2020.116621), which requires defining a seed region to select rsfMRI volumes that exceed a user‐defined BOLD signal threshold within that region. The toolbox also includes a seed‐free option that considers all volumes regardless of regional activity. For this study, we opted for the seed‐free method to capture whole‐brain coactivation states. Clustering was performed at the voxel‐wise level.

For each participant and fMRI volume, data were first masked using a whole‐brain mask to remove noisy voxels and reduce computational load. This step excluded regions such as cerebrospinal fluid that may exhibit spurious BOLD fluctuations capable of biasing the clustering procedure. For each volume, a vector of BOLD signal values across all voxels was then generated, producing a data matrix for each subject. These matrices were concatenated across subjects to create a group‐level dataset for clustering. *K*‐means clustering was applied to classify the data into *k* coactivation states. Candidate solutions were evaluated for *k* values ranging from 5 to 10. For each *k*, the clustering procedure was repeated 20 times using a randomly selected subsample of 80% of the fMRI volumes to ensure stability of the solution [18]. To guide the selection of the optimal number of clusters, we computed the Silhouette and Calinski–Harabasz indices. Both metrics produced inconsistent recommendations and tended to underestimate the number of CAPs, identifying an optimal solution at *k* = 2. Therefore, following the approach described by Liu et al. [18], we visually compared the CAP maps obtained across candidate solutions and selected the final value of *k* based on the interpretability and separability of the resulting patterns. The number of clusters was set to *k* = 6 because it represented the highest value producing clearly distinguishable CAPs without redundancy, whereas lower values generated too few distinct patterns and limited the variability of CAP states.

Labeling of each CAP into a brain network was performed using Component Labeler, a tool provided by GIFT toolbox [25] (Supporting Information 1: Figure S1), to analyze how closely a CAP resembles established brain networks. Each CAP was correlated with a given template (in this study, resting‐state network [RSN] atlas provided by GIFT) computing a correlation index (*r*) using cosine similarity method [26]. The absolute value of *r* reflects the degree of similarity between a CAP and a specific network, with higher values indicating a better match. The resulting CAPs represented activity in the ventral (CAP‐1), dorsal DMN (CAP‐2 and CAP‐5), visuospatial network (VSN; CAP‐3 and CAP‐6), and right executive control network (ECN; CAP‐4).

**Figure 1:**
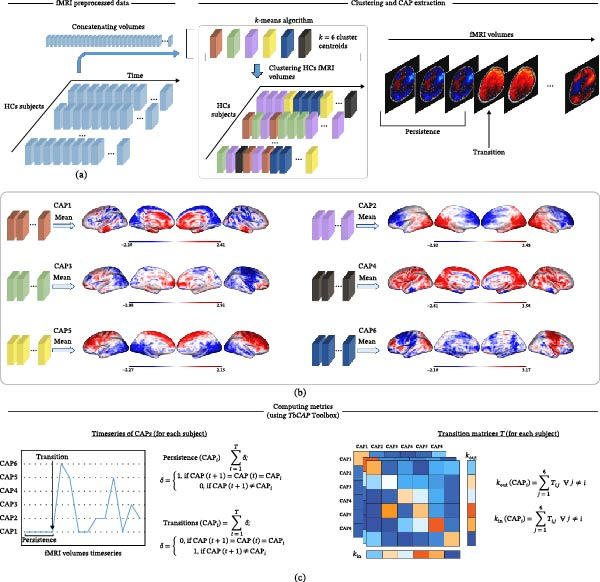
Pipeline for evaluating coactivation patterns (CAPs) and metrics dynamics: (a) for each healthy control participant we masked each volume using a whole‐brain mask, convert to *Z*‐scores and create a data vector of concatenated volumes to perform *k*‐means using *k* = 6. (b) Clustering process classified fMRI volumes in six states and then we averaged those assigned to the same cluster to compute CAPs. CAPs were then assigned to patients based on their spatial similarity. At the end of this step, we obtain time series for each participant with each volume categorized in a CAP. (c) We determine metrics as follows: persistence (for each CAP), defined as the volume to volume maintenance in a same CAP (or state); transistion (for each CAP) as the number of volumes changing state to any other. To obtain metrics in/out degree, we first computed with TbCAP toolbox the transition matrix *T* for each subject containing the number of transitions between two possible states: for a particular CAP_*i*_, in‐degree is computed as the sum along *i*‐th row and out‐degree the sum along *i*‐th column (excluding diagonal elements), both normalized for number of volumes.

For all subjects, we obtain metrics of CAP dynamics using Matlab custom code: (1) persistence that is the total state‐to‐state maintenance in a specific brain state and (2) transitions defined as total frequency of state‐to‐state transitions from one volume to the next (e.g., frequency to moving from State A to State B). Using TbCAPs we added measure of (3) in‐degree (*k*_in_), defined as how likely a CAP is visited from any other, and (4) the out‐degree (*k*_out_)—how likely a CAP is exited towards any other. The workflow for generating CAPs is displayed in Figure 1.

These CAP metrics are indicative of RSN engagement, offering insight into which RSNs are active, for how long, and how they interact overtime. This can facilitate a deeper understanding of brain network function and dysfunction due to tumor presence.

### 2.6. Statistical Analysis

Differences in CAP metrics between patients with left vs. AD language and patients with vs. without postoperative aphasia were studied with *t*‐test. For the purposes of this analysis, all patients with any type of aphasia were categorized together as “aphasic,” regardless of subtype (0 = no deficit and 1 = aphasia of any kind). Statistical significance level for multiple comparison was corrected to reduce false positives using Benjamini Hochman correction (significance at *p*  < 0.05). To test the effect of tumor volume, tumor type (HGG, LGG, and metastases), and location on CAP metrics we employed ANOVA with significance set at *p*  < 0.05. We included tumor locations with sufficient representation: frontal (*n* = 39), parietal (*n* = 24), and temporal (*n* = 26). Locations with low subject numerosity were excluded to avoid class imbalance. To test the relationship between tumor location and language network dominance, we performed an additional ANOVA with significance set at *p*  < 0.05 including the hemispheric LI obtained from task‐based fMRI. To evaluate the clinical predictive value of the imaging measures, we performed logistic regression analyses to predict postoperative aphasia. Two models were tested (1): a model including CAP metrics together with the language LI and (2) a model including the LI alone.

## 3. Results

### 3.1. Participants

**Figure 2:**
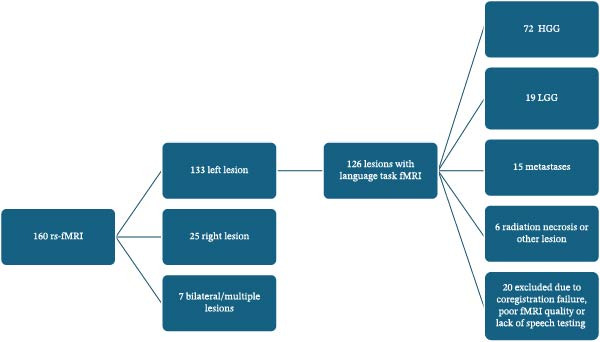
Flowchart of patient recruitment.

106 patients were recruited (72 high‐grade gliomas—HGG 26F, 19 low‐grade gliomas—LGG 8F, 15 metastases, 43F, age 61.15 ± 8.95). A flowchart of patient selection is reported in Figure 2. Patients demographics and tumor characteristics are available in Table 1. Tumor distribution is shown in Supporting Information 2: Figure S2. Thirty‐two patients demonstrated post‐operative aphasia as defined in our methods. A distribution of speech deficits across patients before and after surgery is represented in Supporting Information 3: Figure S3.

**Table 1:** Characteristics of brain tumor patients.

Brain tumor patients			
Subjects	106		
Age	61.15 ± 8.95		
Gender			
Female	43		
Male	63		
Tumor grade			
Low grade (LGG)	19		
High Grade (HGG)	72		
Metastasis	15		
Tumor localization			
Temporal	26		
Cerebellum	0		
Insula	10		
Frontal	39		
Occipital	4		
Parietal	24		
Central	3		

**Lateralization**	**LD**	**AD**	**n.a.**

Hemispheric	62	40	4
Frontal	66	36	4
Broca	70	32	4
Temporal	49	53	4
Wernicke	45	57	4
Cerebral	64	38	4

### 3.2. CAP Analysis

A total of six CAPs were characterized. CAP1 corresponds to the ventral DMN deactivation (*r* = −0.1244), involving mainly left and right cuneus, precuneus, and anterior cingulate cortex; CAP2 corresponds to the dorsal DMN deactivation (*r* = −0.3039), mainly located in the posterior cingulate and medial prefrontal cortex, CAP5 corresponds to the dorsal DMN activation (*r* = 0.2297); CAP3 and CAP6 include parts of the VSN deactivation (*r* = −0.126) and activation (*r* = 0.101), respectively, such as the intraparietal sulcus and frontal eye fields; CAP4 corresponds to components of the ECN activation (*r* = 0.145) such as the dorsal–lateral prefrontal cortex and parietal cortex. A dynamic representation of patients’ CAPs in the different groups is displayed in the Supporting Information 4: Video 1–Supporting Information 7: Video 4.

**Figure 3:**
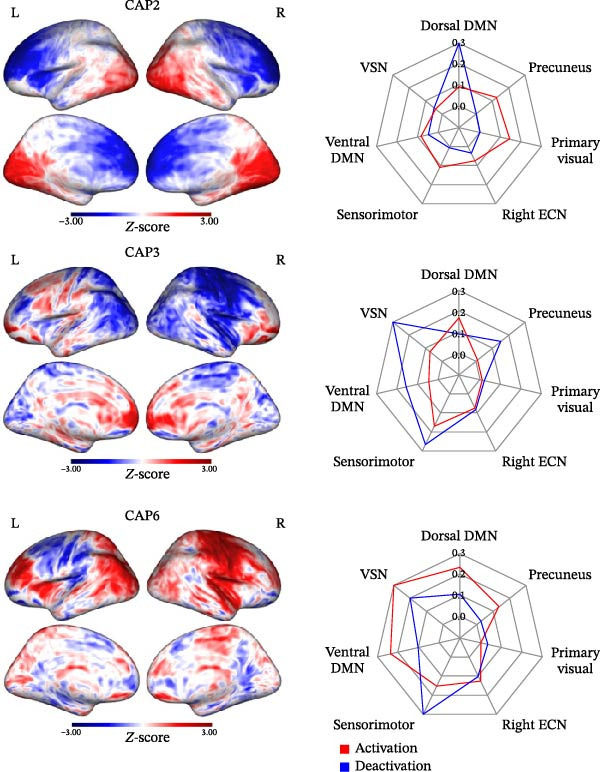
Visual representation of the significant coactivation patterns (CAPs) identified in this study and relative network overlap.

**Figure 4:**
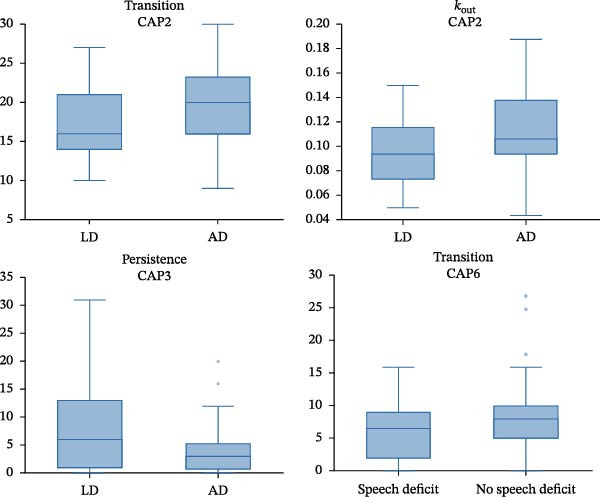
Boxplots representing the significant results in coactivation patterns (CAPs) 2, 3 and 6. CAP2 showed increased transition and out‐degree in patients with atypical language dominance. CAP3 showed lower persistence in patients with atypical language dominance. CAP6 showed increased transition in patients with better speech performance.

For temporal LI, AD patients showed higher out‐degree and transitions in CAP2 (dorsal DMN; *p* = 0.004, *p* = 0.006) and lower persistence in CAP3 (VSN; *p* = 0.002) compared to LD patients. Patients without speech deficit after surgery demonstrated higher transitions in CAP6 (VSN; *p* = 0.024). The remaining CAPs did not produce statistically significant results in the comparison LD vs. AD. Figure 3 depicts significant CAPs. The significant results are presented in the form of boxplots in Figure 4. The results are also reported in Supporting Information 8: Tables S1 and S2.

**Figure 5:**
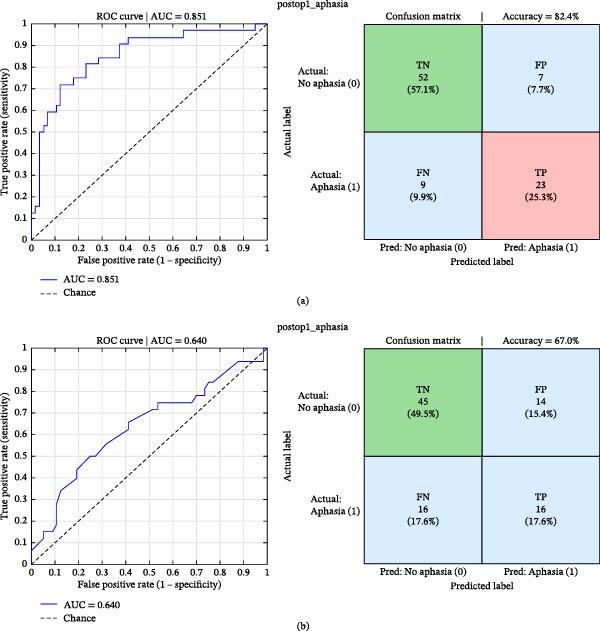
Prediction of postoperative aphasia using logistic regression models. Receiver operating characteristic (ROC) curves and corresponding confusion matrices illustrating the performance of two predictive models. (a) Model including CAP metrics together with the language laterality index. (b) Model including the language laterality index alone. The combined model demonstrates higher discriminative performance, with a larger area under the curve (AUC) and improved classification metrics compared with the model based solely on laterality index. Confusion matrices show the distribution of true and predicted outcomes for postoperative aphasia in each model.

The combined model including CAP metrics and LI demonstrated good predictive performance. Receiver operating characteristic analysis yielded an area under the curve (AUC) of 0.851, with an optimal classification threshold of 0.464. Using a classification threshold of 0.517, the model achieved an overall accuracy of 82.4%, sensitivity of 71.9%, and specificity of 88.1%. The positive predictive value was 76.7% and the negative predictive value was 85.2% (Figure 5).

In contrast, the model including only the LI showed lower predictive performance. The ROC analysis yielded an AUC of 0.640 with an optimal threshold of 0.410. At this threshold, the model achieved an overall accuracy of 67.0%, sensitivity of 50.0%, and specificity of 76.3%, with a positive predictive value of 53.3% and a negative predictive value of 73.8% (Figure 5).

No significant differences were found between tumors of different type or volume in any of the CAP metrics. The ANOVA test revealed a slight significant effect of tumor location on transition, in/out degree of CAP2, with parietal tumors showing increased metrics compared to other locations. The additional ANOVA analysis did not reveal any significant associations between tumor location in the left hemisphere and language network dominance.

## 4. Discussion

Our findings reveal a previously unreported pattern in patients with brain tumors affecting the language‐dominant hemisphere: specifically, we observed increased engagement of the VSN in patients without post‐operative speech deficits. This result challenges existing models that primarily emphasize the recruitment of domain‐general systems, such as the CCN and DMN, or contralateral language homologs, and opens up a new line of inquiry into how non‐language‐specific systems contribute to neuroplasticity.

Neurocognitive models of cross‐modal compensation provide a useful framework for interpreting this phenomenon. When a primary cognitive system is compromised, such as the language network in our tumor patients, the brain may recruit other systems, often from different modalities, to support the impaired function. This has been well documented in sensory deprivation studies, where the dorsal visual stream in blind individuals is repurposed to process auditory or tactile inputs, enabling spatial attention and navigation via non‐visual cues [27, 28].

Our results suggest that a similar mechanism may be at play in brain tumor patients, particularly those exhibiting functional reorganization without significant post‐surgical speech deficits (suggestive of adaptive plasticity). The VSN, though not classically associated with language, may provide spatial–attentional scaffolding that facilitates access to and retrieval of language functions under conditions of cortical disruption. This hypothesis complements the recent models of reorganization that emphasize: (i) increased interhemispheric recruitment of contralateral homologs, (ii) enhanced interaction between functionally distinct brain networks, and (iii) compensatory engagement of domain‐general systems when specialized functions are compromised [29]. Interestingly, the observed engagement of VSN may suggest mechanisms similar to those involved in sign language processing, where visual and attentional cues play a key role in facilitating linguistic encoding and retrieval [30, 31]. In this context, VSN activation may enhance functional compensation for damaged language areas by integrating visual, attentional, and semantic cues, especially in tasks requiring multimodal language processing.

In addition to the engagement of VSN, we also observed a distinct pattern in patients with atypical language dominance, a surrogate marker of language reorganization. These individuals showed increased transitions in the DMN deactivation, regardless of whether they presented with pre‐ or postsurgical aphasia. Such finding reflects a more frequent engagement of the suppressed state (deactivation) of the DMN. It is known that brain tumors decrease suppression of the DMN during task performance [32], which is considered in line with the disruption of cognitive processing typical of this pathology. DMN deactivation is indeed pivotal for task performance [33]. Our findings suggest that effective reorganization may rely on a strengthened capacity to deactivate the DMN.

Evidence from poststroke aphasia recovery shows that domain‐general networks such as cognitive control become more active after the injury and are associated with better language outcomes [34, 35]. The relationship between DMN and CCN is one of dynamic, often anticorrelated interaction; the DMN is focused on internal mentation, semantic processing, and integration of autobiographical and conceptual knowledge [36], and typically deactivates when cognitive control is required to focus on an external task [37]. Gordon et al. [38] further demonstrated that the DMN couples with other large‐scale systems, including language and CCN, through specific subnetworks identified with resting‐state fMRI. Such dynamic coupling may underpin high‐level cognition, including language comprehension and production [38]. In fact, tumor studies have linked CCN engagement with better speech performance [13]. In this light, our findings may capture the complementary side of this process: increased DMN suppression facilitates CCN activity, creating a favorable context for language reorganization in the setting of brain tumors.

Our study advances current understanding by showing that while atypical dominance is associated with stronger DMN suppression, only VSN dynamics are directly linked to the absence of postoperative speech deficits. These results refine prior literature and suggest a layered model of language reorganization: (i) DMN suppression may enable interhemispheric reorganization and right‐hemispheric recruitment, possibly by empowering cognitive control processes, but (ii) this alone may not guarantee full functional compensation. In contrast, VSN engagement may provide the necessary integration of visuospatial and attentional mechanisms to support adaptive strategies for language encoding and retrieval.

To evaluate the clinical predictive value of the imaging measures, we performed logistic regression analyses to predict postoperative aphasia. We compared two models: one including CAP metrics together with the language LI and another including the LI alone. The model integrating CAP metrics demonstrated substantially better predictive performance than the model based solely on language laterality, indicating that CAP–derived measures provide additional predictive information beyond conventional task‐based lateralization metrics.

This observation is consistent with prior literature. Although shifts in language dominance have been widely reported in patients with brain tumors and are often interpreted as evidence of interhemispheric reorganization [3], changes in laterality alone do not necessarily reflect compensatory functional mechanisms. Network‐level measures derived from functional connectivity provide a broader description of large‐scale brain organization and have been shown to better capture the clinical significance of reorganization processes than task‐based lateralization metrics alone [5]. In this context, CAP dynamics may offer complementary information about the stability and interaction of functional networks that is relevant for predicting postoperative language outcomes.

Together, our findings offer evidence for a distributed and flexible compensatory architecture that draws on both domain‐general (e.g., DMN) and modality‐specific (e.g., VSN) resources in response to cortical disruption by tumors. Clinically, they suggest that preoperative CAP analysis could offer predictive insight into which patients exhibit compensatory language reorganization, potentially undergoing radical surgery with no postoperative deficits. Future research should explore whether visuospatial engagement can be enhanced through prehabilitation which could include specific cues targeted to this network. In this view, visuomotor tasks, possibly including sign language models, may enhance adaptive language plasticity before and after surgery.

This study has some limitations. While we relied on language dominance and the presence of aphasia as clinical indicators, future studies would benefit from a more comprehensive cognitive testing to better validate the relationship between CAP dynamics and other brain functions. Second, while our goal was to examine patterns of language reorganization across tumor types, we did not perform separate group analyses. Instead, we used an ANOVA including tumor size, type, and location to test their effects on CAPs. Although this approach allowed us to account for these clinical variables, the overall sample size limited the power to detect more subtle or interaction effects. In particular, the subgroup of patients with metastases was relatively small, which constrains the interpretability of findings within this cohort. This reflects our inclusion criteria, which required single and well‐defined lesions to minimize confounding effects in the network analysis. Prospective studies with larger and more stratified cohorts are needed to confirm and extend these findings.

## 5. Conclusion

Our findings reveal that adaptive language reorganization in brain tumor patients involves not only domain‐general networks but also increased engagement of the VSN, suggesting a cross‐modal compensatory mechanism. These insights, made possible through CAP analysis, highlight the brain’s dynamic adaptability and may help guide surgical planning and personalized interventions to preserve language function.

**Nomenclature**
BOLD: Blood‐oxygenation level dependent
CAP: Coactivation patterns
CCN: Cognitive control network
DMN: Default mode network
GIFT: Group independent component analysis of fMRI toolbox
HGG: High‐grade glioma
LGG: Low‐grade glioma
MNI: Montreal Neurological Institute
VSN: Visuospatial network.

## Funding

This research was supported by the American Roentgen Ray Society (ARRS) Scholarship 2025 (Luca Pasquini).

## Disclosure

Preliminary results of this work have been presented at the American Society of Neuroradiology (ASNR) 63rd annual meeting, May 17−21, 2025, Philadelphia, PA.

## Ethics Statement

This study was approved by the Institutional Review Board, and the requirement for informed consent was waived. All procedures were conducted in accordance with the Declaration of Helsinki.

## Conflicts of Interest

Andrei I. Holodny is the owner/president of fMRI Consultants, LLC, a purely educational entity. Luca Pasquini is the owner/president of Panavem LLC. The other authors declare no conflicts of interest.

## Supporting Information

Additional supporting information can be found online in the Supporting Information section.

## Supporting information


**Supporting Information 1** Figure S1: Representation of the network atlas labeling on inflated brain.

**Supporting Information 2** Figure S2: Tumor lesion overlap map summarizing tumor locations across patients.

**Supporting Information 3** Figure S3: Distribution of language deficits across patients.

**Supporting Information 4** Video 1: Dynamic representation of mental states of patients with atypical language dominance characterized through the alternation of coactivation patterns.

**Supporting Information 5** Video 2: Dynamic representation of mental states of patients with left hemispheric dominance characterized through the alternation of coactivation patterns.

**Supporting Information 6** Video 3: Dynamic representation of mental states of patients with no speech deficits characterized through the alternation of coactivation patterns.

**Supporting Information 7** Video 4: Dynamic representation of mental states of patients with aphasia characterized through the alternation of coactivation patterns.

**Supporting Information 8** Table S1: Significant values for CAP metrics in language reorganization estimated through shift in lateralization from left to atypical dominance (temporal LI). Table S2: Significant values for CAP metrics in postsurgical aphasia.

## Data Availability

The data supporting the findings of this study are available from the corresponding author upon reasonable request.
