# Supplementary figures and images for: Resting‐State Coactivation Patterns of Language Reorganization in Brain Tumors
Source: Neural Plast. 2026 May 21;2026:1421115. doi: 10.1155/np/1421115 (PMC13195184; doi:10.1155/np/1421115)

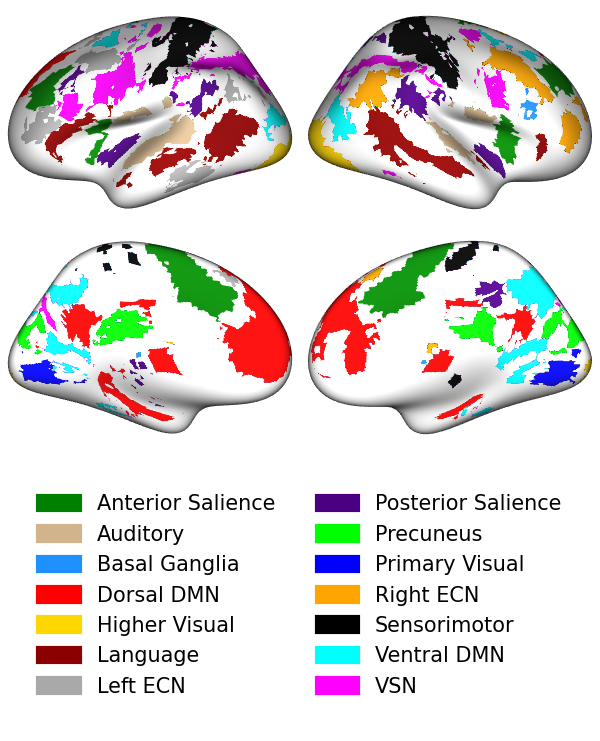

Supplement: Supplementary file 1 — Supporting Information 1 Figure S1: Representation of the network atlas labeling on inflated brain. [file NP-2026-1421115-s006.png]

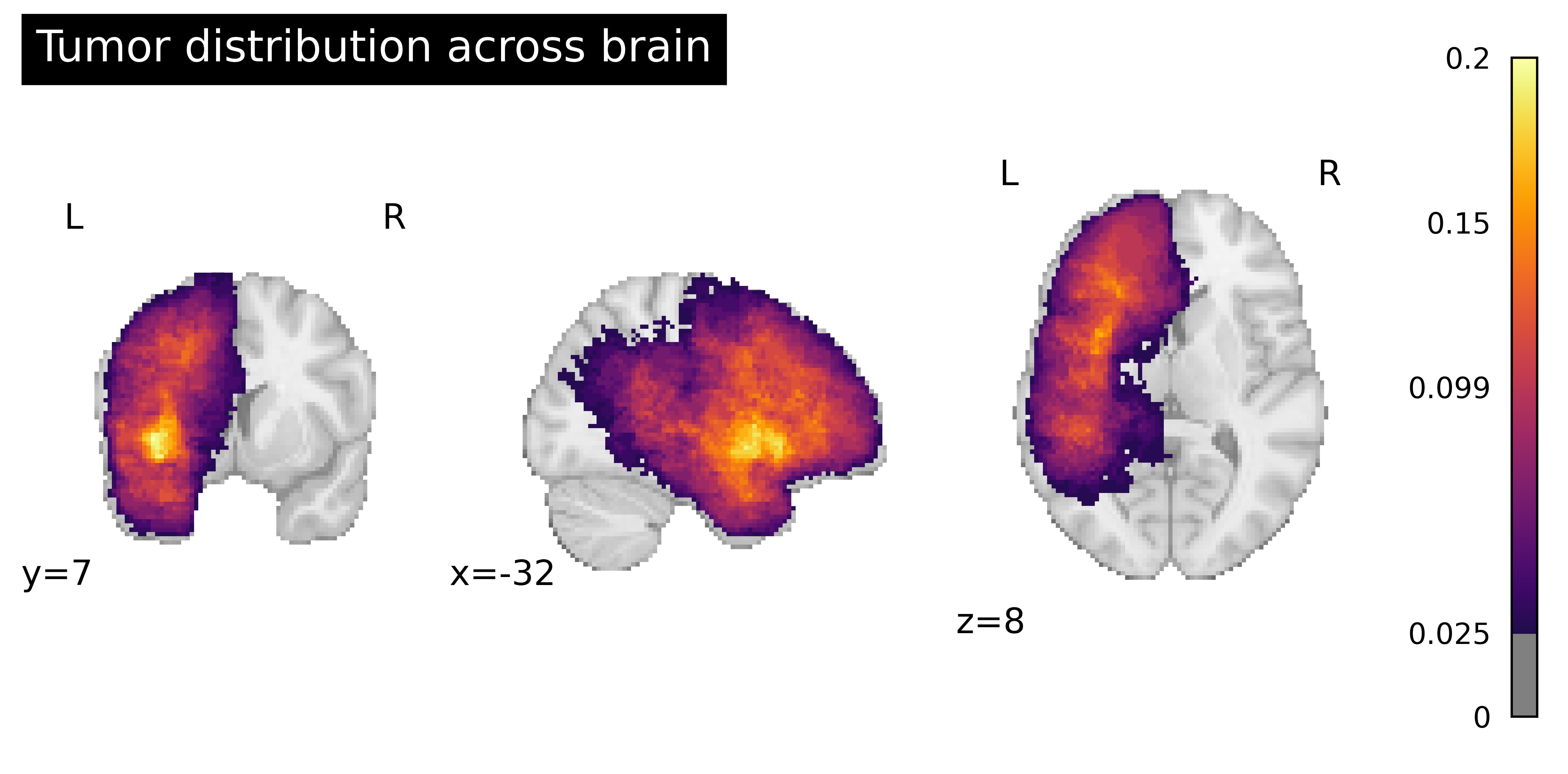

Supplement: Supplementary file 2 — Supporting Information 2 Figure S2: Tumor lesion overlap map summarizing tumor locations across patients. [file NP-2026-1421115-s005.png]

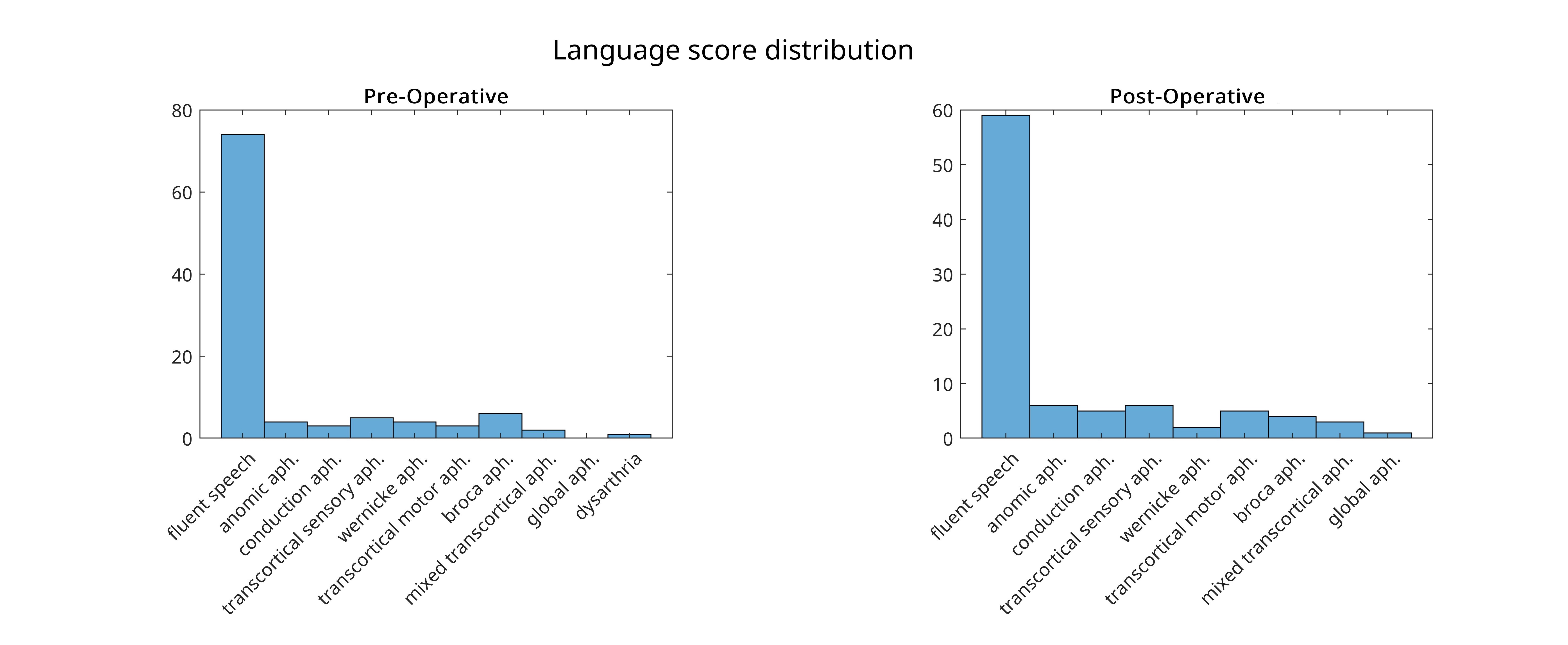

Supplement: Supplementary file 3 — Supporting Information 3 Figure S3: Distribution of language deficits across patients. [file NP-2026-1421115-s004.jpg]
