# Supplementary material for: Resting‐State Coactivation Patterns of Language Reorganization in Brain Tumors
Source: Neural Plast. 2026 May 21;2026:1421115. doi: 10.1155/np/1421115 (PMC13195184; doi:10.1155/np/1421115)
Supplement: Supplementary file 8 — Supporting Information 8 Table S1: Significant values for CAP metrics in language reorganization estimated through shift in lateralization from left to atypical dominance (temporal LI). Table S2: Significant values for CAP metrics in postsurgical aphasia. [file NP-2026-1421115-s002.docx]

|  |  | Transitions | | Persistence | | Kout | |
| --- | --- | --- | --- | --- | --- | --- | --- |
|  |  | p | CI | p | CI | p | CI |
| ***CAP2*** | PCC / MPFC (Dorsal Default Mode Network) | 0.0062 | [-4.5544; -0.7733] |  | | 0.0045 | [-0.0279; -0.0053] |
| ***CAP3*** | Intraparietal Sulcus / Frontal Eye Fields (Visuospatial Network) |  | | 0.0028 | [1.3066; 6.0781] |  | |

**Table S1**. Significant values for CAP metrics in language reorganization estimated through shift in lateralization from left to atypical dominance (temporal LI).

**Table S2**. Significant values for CAP metrics in post-surgical aphasia.

|  |  | Transitions | |
| --- | --- | --- | --- |
|  |  | p | CI |
| ***CAP6*** | Intraparietal Sulcus / Frontal Eye Fields (Visuospatial Network) | 0.0245 | [-4.5962; -0.3244] |
